# The Organization of the Primate Insular Cortex

**DOI:** 10.3389/fnana.2019.00043

**Published:** 2019-05-08

**Authors:** Henry C. Evrard

**Affiliations:** ^1^Functional and Comparative Neuroanatomy Laboratory, Werner Reichardt Center for Integrative Neuroscience, Tübingen, Germany; ^2^Max Planck Institute for Biological Cybernetics, Tübingen, Germany

**Keywords:** interoception, autonomic nervous system, emotion, cognition, awareness, architectonics, tract-tracing

## Abstract

Long perceived as a primitive and poorly differentiated brain structure, the primate insular cortex recently emerged as a highly evolved, organized and richly connected cortical hub interfacing bodily states with sensorimotor, environmental, and limbic activities. This insular interface likely substantiates emotional embodiment and has the potential to have a key role in the interoceptive shaping of cognitive processes, including perceptual awareness. In this review, we present a novel working model of the insular cortex, based on an accumulation of neuroanatomical and functional evidence obtained essentially in the macaque monkey. This model proposes that interoceptive afferents that represent the ongoing physiological status of all the organs of the body are first being received in the granular dorsal fundus of the insula or “primary interoceptive cortex,” then processed through a series of dysgranular poly-modal “insular stripes,” and finally integrated in anterior agranular areas that serve as an additional sensory platform for visceral functions and as an output stage for efferent autonomic regulation. One of the agranular areas hosts the specialized von Economo and Fork neurons, which could provide a decisive evolutionary advantage for the role of the anterior insula in the autonomic and emotional binding inherent to subjective awareness.

## Introduction

The forebrain representation and integration of the physiological condition of the organs and tissues of the body (interoception) shapes perceptual awareness and underlies the neurobiology of subjective feelings (Craig, 2009; Critchley and Garfinkel, 2018). The neuronal pathway that encodes interoception from the periphery to the spinal cord, brainstem, thalamus, and cerebral cortex does not primarily end in the classical primary somatosensory cortex but in the dorsal fundus of the insular cortex, or “primary interoceptive cortex” (Craig, 2002). Functional evidence in humans indicates that the primary interoceptive cortex hosts a rather “objective” topographic representation of physiological changes (e.g., linear innocuous cooling or linear transcutaneous histamine concentration), which translates into a “subjective” representation in the anterior insular cortex (AIC) where brain activity tends to correlate with the perceptual report of a sensation rather than with the actual physiological changes itself (Craig et al., 2000; Drzezga et al., 2001; Craig, 2009). In fact the activity of the human AIC strongly correlates with a vast range of subjective activities, including subjective bodily sensations (Mutschler et al., 2009), emotional feelings [e.g., (Bartels and Zeki, 2004; Gu et al., 2013; Smith et al., 2015)], empathy (Lamm and Singer, 2010), as well as more complex perceptions, such as, the recognition of oneself in a mirror (Devue and Bredart, 2011) and other visual perceptual tasks (Salomon et al., 2016), temporal discrimination (Pastor et al., 2004), and intention forming (Brass and Haggard, 2010). The AIC, together with the anterior cingulate (ACC), is also the cortical region with the highest concentration of the specialized von Economo (VEN) and Fork (FN) neurons (Allman et al., 2010). Both neurons are selectively depleted in the early stage of the behavioral variant of the frontotemporal dementia (bvFTD), which is characterized by a subtle loss of self-conscious feelings (Kim et al., 2011; Santillo and Englund, 2014; Nana et al., 2019). Unsurprisingly, the human AIC and ACC are the most common targets of psychiatric disorders (Nagai et al., 2007; Seeley, 2008; Goodkind et al., 2015). Furthermore, a single-case clinical report indicated that microstimulation of the AIC/claustrum region can immediately alter states of consciousness (Koubeissi et al., 2014) and its functional connectivity with the arousal centers of the brainstem has been shown to be massively disrupted in coma (Fischer et al., 2016). More recently, the AIC was also shown to be temporally the last commonly active brain region in response to functionally distinct sensory stimuli (words, touch, and pain) during the anesthetic induction of a complete loss of behavioral response (Warnaby et al., 2016), suggesting a role in gating salient information in their access to conscious behavior. Although its exact role still has to be elucidated, the insula, and in particular its anterior part, are likely to have a crucial role in functions that directly relate to awareness. In order to unravel fundamental organizational principles, our laboratory recently initiated a vast series of neuroanatomical, functional, and molecular examinations of the macaque monkey insula. The present review incorporates findings from our and other laboratories into a novel model of the anatomical and functional organization of the insular cortex, upon a backdrop of refined architectonic parcellation.

## Macroscopic Organization

**Figure 1:**
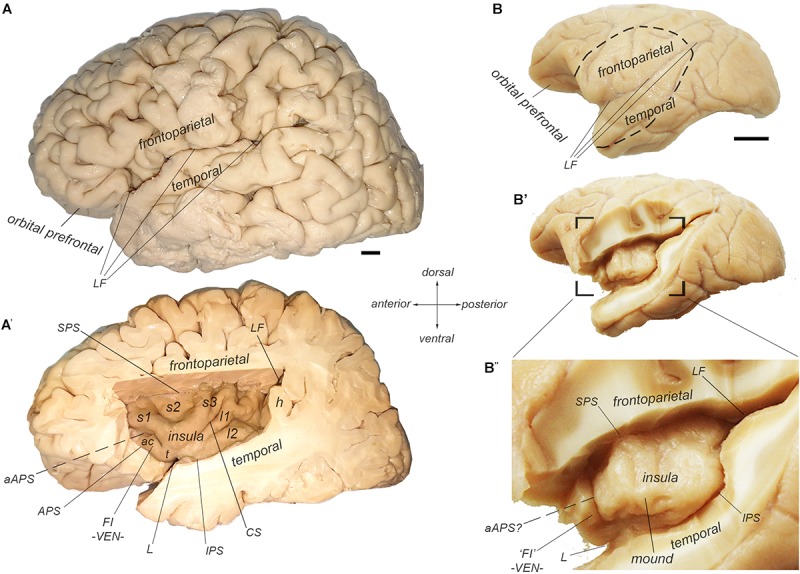
Morphological delineation of the insula and its main structural landmarks in the human and cynomolgus macaque monkey brains. **(A**,**A’)** Photographs of the lateral (left) aspect of a human brain with **(A)** and without **(A’)** the frontoparietal and temporal opercula that cover the insular cortex, which is located in the depth of the LF. The human insula contains two posterior long posterior gyri (l1 and l2) separated from three anterior short gyri (s1-3) by the central sulcus of the insula (CS). The anterior insula contains an additional accessory short gyrus, which varies in size and demarcation. We consider that this accessory gyrus overlaps with the so-called FI, which is characterized by its high concentration of von Economo neurons (VEN). The insula is delimited by the superior (SPS), inferior (IPS), and anterior (APS) peri-insular sulci. “h” denotes one of the temporal gyri of Heschl. **(B**–**B”)** Photographs of the lateral (left) aspect of a cynomolgus macaque monkey brain with **(B)** and without **(B’**,**B”)** the frontoparietal and temporal opercula that cover the insular cortex, which is located in the depth of the LF. The macaque insula is essentially smooth with an incipient ventral gyrus or “mound” and a shallow, but distinct anterior sulci or “ridge,” which marks the limit of the anterior insular region containing the VEN, and which we consider a structural homolog of the human FI.

In anthropoid primates, the insular cortex constitutes a separate cortical lobe, located on the lateral aspect of the forebrain, in the depth of the Sylvian or lateral fissure (LF) (Figure 1; Reil, 1809; Retzius, 1902; Naidich et al., 2004; Afif and Mertens, 2010; Gonzalez-Arnay et al., 2017). (See also Naidich in this special issue). It is adjoined anteriorly by the orbital prefrontal cortex, and it is covered dorsally by the frontoparietal operculum and ventrally by the temporal operculum (Figure 1A,B). The excision of the two opercula and part of the orbital prefrontal cortex reveals the insula proper, delimited by the anterior, superior, and inferior peri-insular (or limiting or circular) sulci (Figure 1A’).

The human insula is gyrencephalic (i.e., convoluted), like the rest of the human neocortex (Figure 1A’). It is divided into posterior and anterior lobules by the central sulcus of the insula (CS). There are usually two long (*longus*) gyri in the posterior lobule (l1 and l2), three short (*brevis*) anterior gyri in the anterior lobule (s1, s2, and s3), and one accessory gyrus (ac), which is continuous with the ventral transverse gyrus (t). The orientation and size of the gyri as well as the exact number of short gyri varies across individuals and hemispheres (Wysiadecki et al., 2018). The accessory gyrus varies in volume and demarcation (Ture et al., 1999; Wysiadecki et al., 2018). When large, it is separated from the anterior short gyrus by a distinct folding (named here “accessory APS”, aAPS) that bifurcates from the actual APS (Ture et al., 1999). Notably, the localization of the accessory gyrus and transverse gyrus corresponds approximately with the localization of the frontoinsula (FI) (Von Economo and Koskinas, 1925) which was recently defined by the presence of a high concentration of VENs and FNs (von Economo, 1926; Allman et al., 2010).

The macaque insula is almost entirely lissencephalic (i.e., smooth), which complicates establishing homologies solely based on macroscopic criteria (Figure 1B”). The macaque insula has an incipient horizontal gyrus (or “mound”) ventrally, and, in many cases, a shallow but distinct vertical ridge anteriorly. The cortical region anterior to this vertical ridge contains a high concentration of VENs and FNs (Evrard et al., 2012), suggesting a partial structural homology of this region with the human FI, and of the vertical ridge *per se* with the human accessory APS. Unlike in humans, beyond this shallow dimple, there is no distinct APS separating the insula from the orbital prefrontal cortex; they are rather continuous.

## Architectonic Parcellation

**Figure 2:**
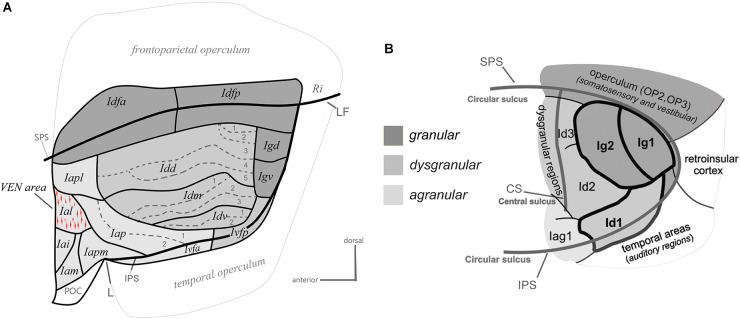
Architectonic parcellation of the insular cortex in the macaque monkey **(A)** and human **(B)**. Both panels present a schematic flat map view of the insula, adapted with permission from Kurth et al. (2010a) and Evrard et al. (2014). The human flat map shows only the posterior lobule of the insula. In both maps, the granular, dysgranular, and agranular sectors are represented by different shades of gray (see scale), with non-insular regions shaded a lighter gray than the agranular insula. See the main text for a full description.

One major step in examining the organization of a brain region beyond its gross anatomy is to establish its architectonic parcellation. This is typically based on the microscopic examination of local variations in cellular (cytoarchitecture) and/or fibrous (myeloarchitecture) organization patterns in thin (tens of microns) brain sections colored with classical histological staining methods or standard immunohistochemical methods (Brodmann, 1909; Vogt and Vogt, 1919; Carmichael and Price, 1994). Using primarily cytoarchitectonic analyses, prior authors divided the primate insula into posterior “granular,” intermediate “dysgranular,” and anterior “agranular” sectors, based on the presence, thickness and distinctiveness of Meynert’s granule cell layers 4 and, to some extent, 2 (Brodmann, 1909; von Bonin and Bailey, 1947; Roberts and Akert, 1963; Sanides, 1968; Burton and Jones, 1976; Mesulam and Mufson, 1982a; Gallay et al., 2012; Morel et al., 2013). In a recent cyto- and myelo-architectonic re-examination of the macaque monkey insula, we observed that each of these classical sectors consistently contains smaller and sharply delimited areas (Figure 2A; Evrard et al., 2014), which could correlate with the vast diversity of specific functions and disorders that the insula is involved with.

The granular sector of the insula contains four areas including one anterior area (Idfa) and one posterior area (Idfp) straddling the fundus of the SPS, and two areas in the posterior end of the insula, one dorsal (Igd) and one ventral (Igv). The dysgranular sector contains four areas. They are the dorsal (Idd), mound (Idm), ventral (Idv), and posterior ventral fundal (Ivfp) dysgranular areas. The mound area Idm is contained within and makes up most of the ventral mound observed in the macroscopic examination of the gross morphology of the macaque insula. Three of the dysgranular areas (Idd, Idm, and Idv) contain three to five smaller architectonic subareas. These subareas form together a series of “insular stripes” that are orientated horizontally, in parallel with the dorsal fundus areas Idfa and Idfp. Finally, the anterior agranular sector contains seven areas. Two areas are located posterior to the limen (L): the posterior agranular area (Iap), which contains two subareas, and the anterior ventral fundal area (Ivfa), which prolongs the dysgranular Ivfp anteriorly. The five other agranular areas (Iam, Iapm, Iai, Ial, and Iapl) are located anterior to the limen, mostly in the putative homolog of the human FI, except for Iapm and Iapl, which cross the shallow vertical ridge thought to define the posterior limit of the putative homolog of the FI. Notably, in the macaque monkey, the VEN and FN occur in a small cluster (Evrard et al., 2012) that corresponds likely to the Ial (Horn and Evrard, unpublished), which was defined independently prior to the discovery of the VEN and FN in macaque, based only on low-magnification architectonic criteria (Evrard et al., 2014). The five agranular areas anterior to the limen are mostly identical to the agranular insular areas defined earlier by Carmichael and Price (1994) in their examination of the orbital prefrontal cortex. Recent observations in our laboratory suggest, however, a small adjustment of the definition of some of their original areas (see below). Other authors have grouped these areas together in “periallocortex” and “proisocortex” areas, considering them a part of the caudal end of the orbital prefrontal cortex, just posterior to area 13 (Barbas and Pandya, 1989)^1^. As shown in detail in the next section, although complex, our novel architectonic map offers a clear and explanatory structural *Bauplan* for the examination of the connectivity, and functional organization of the insula.

Various parcellation schemes have been proposed for the human insula since the original work of Brodmann (Vogt, 1911; Rose, 1928; Brockhaus, 1940; Morel et al., 2013). Our map in the macaque monkey bears basic resemblance with some of the earliest maps by Rose (Rose, 1928) and Brockhaus (Brockhaus, 1940), as discussed in detail elsewhere (Evrard et al., 2014). Most recently, the groups of Amunts and Zilles used computerized measurements of laminar optical density on the posterior lobule of the human insula (Eickhoff et al., 2006; Kurth et al., 2010a; Figure 2B). The authors reported so far: one “opercular” area (OP3) in the posterior end of the SPS, two granular areas (Ig1 and Ig2), two dysgranular areas extending horizontally anterior to the granular areas (Id2 and Id3), one dysgranular area in the fundus of the inferior peri-insular sulcus (Id1), and one agranular area (Iag1) anterior to Id1 and Id2. The spatial arrangement of the areas in the posterior lobule of the human insula closely resembles the architectonic parcellation of the posterior end of the macaque insula. In both species, there is one distinct granular area in the posterior end of the SPS, two granular areas more ventral in the posterior insula (Igd and Igv), at least two dysgranular areas, anterior to Igd and Igv, that extend longitudinally, and one dysgranular area in the fundus of the IPS that is continuous with a more anterior agranular area. Thus, OP3 could correspond to Idfp; their homology is supported by the observation that both areas are somatotopically organized anteroposteriorly for the representation of interoceptive inputs (Hua et al., 2005; Eickhoff et al., 2007; Bjornsdotter et al., 2009; Craig, 2010; Segerdahl et al., 2015), and both areas are difficult to activate with discriminative tactile stimulation (Craig, 2014). Although this should be verified with direct architectonic comparisons, Ig1 and Ig2 could correspond to Igv and Igd. Id3 and Id2 could correspond to Idd and Idm, respectively, and Id1 could correspond to Idv and Ivfp put together.

The architecture of the anterior lobule of the human insula remains to be mapped. A volumetric comparison made across 30 primate species indicates that the human anterior insula, including the FI, grew disproportionally more than the rest of the insula and the cerebral cortex, in humans compared to all other primate species (Bauernfeind et al., 2013). This disproportionality suggests that the anterior insula may have been recently enlarged and further differentiated in the human brain, as compared to other primate species. The VEN and FN occur in only one architectonic area of the anterior agranular insula in the macaque monkey (Horn and Evrard, unpublished). The recent expansion in humans could have been accompanied by the multiplication of the single VEN area into multiple VEN areas.

## Connectivity and Functional Model

**Figure 3:**
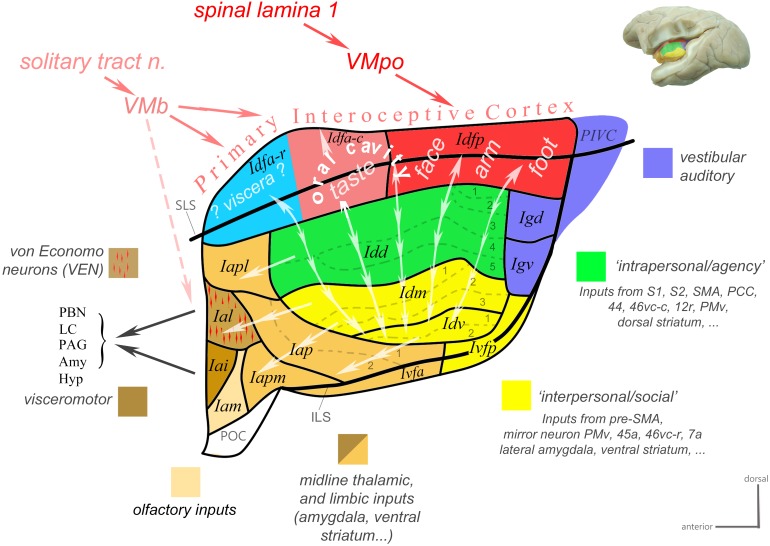
Working functional model of the macaque insula. Color-coded flat map of the insula showing a possible functional organization inscribed within the modular architectonic *Bauplan*. Idfa and Idfp form the “Primary Interoceptive Cortex,” which receives vagal and spinal interoceptive afferents from the solitary tract nucleus and lamina 1 of the spinal cord, via the basal and posterior parts of the ventral medial nucleus of the thalamus (VMb and VMpo), respectively. The sensory interoceptive afferents are somatotopically organized from posterior (foot-to-head, red) to mid (orofacial cavity, pink) to anterior (viscera, blue). The vertical double-ended arrows represent the homotopic transfer of information from the primary interoceptive cortex to the distinct modules of the dysgranular areas. We distinguish two main dysgranular domains: (Craig, 2009) an “intrapersonal” domain (green) integrating interoceptive activity with inputs from cortical and subcortical regions involved in the proprioceptive control of movements; (Critchley and Garfinkel, 2018) an “interpersonal” domain (yellow) integrating interoceptive activity with inputs from cortical and subcortical regions involved in the visuomotor and audiomotor control of movements, in particular when the visual or auditory stimulus relate to a conspecific (e.g., vocalization or facial stimulus). Together with the parieto-insular vestibular cortex (PIVC), the dorsal and ventral granular areas (Igd and Igv; blue) integrate vestibular, auditory, whole-body, and cardiovascular activity; they are likely interconnected with the dysgranular areas. The single-ended arrows represent the final funneling of information from the dysgranular modules to the agranular areas. Most agranular areas, including the VEN area, receive moderate sensory visceral afferents from VMb. At least the VEN area (Ial) and its neighbor (Iai) project directly to the parabrachial nucleus (PBN), and periaqueductal gray (PAG). Adapted with permission from Evrard (2018).

Based on a review of classical tract-tracing data from our and other laboratories as well as of functional studies in the macaque monkey, we recently generated a novel working model of the macaque insula (Evrard and Craig, 2012, 2015; Evrard, 2018). In the present review, we updated this model (Figure 3) and presented more details on the underlying neuroanatomical and functional evidence. This model proposes an integrative flow of interoceptive information processing, from the dorsal granular insula, to the intermediate dysgranular insula, and finally to the anterior agranular insula, structurally bound to the architectonic *Bauplan* described above and functionally inherent with the homeostatic optimization of energy utilization introduced by Craig (2015).

This model begins with a dorsal “primary interoceptive cortex,” formed by Idfp and Idfa, which receives topographically organized spinal and cranial interoceptive afferents via the spinal lamina I (SpL1) and the nucleus of the solitary tract (nTS), and then via the posterior and basal parts of the small, primate-specific ventral median nucleus of the thalamus (VMpo and VMb), respectively (Craig et al., 1994; Blomqvist et al., 2000; Craig, 2002, 2014). It continues with a modular re-representation of the primary interoceptive map across the series of “insular stripes,” in Idd, Idm, and Idv, with each dysgranular stripe putatively integrating interoception with distinct cortical and subcortical multimodal activities. (The exact input/output of each stripe remains to be identified.) Based on the nature of these activities, the dysgranular stripes form, so far, two large groups: one dorsal “intrapersonal” group and one ventral “interpersonal” group, which will be defined in the next paragraphs. Finally, the integrated interoceptive activity is funneled anteriorly from the dysgranular stripes toward the agranular areas. These areas likely act simultaneously, as an additional viscerosensory integrator, a major cortical “output stage” regulating autonomic functions, and a major point of contact with the prefrontal cortex (Krockenberger et al., unpublished), which likely contributes to binding ongoing interoceptive, emotional and cognitive processes.

## Granular Insula

### Dorsal Fundal Granular Areas (Idfp and Idfa)

**Figure 4:**
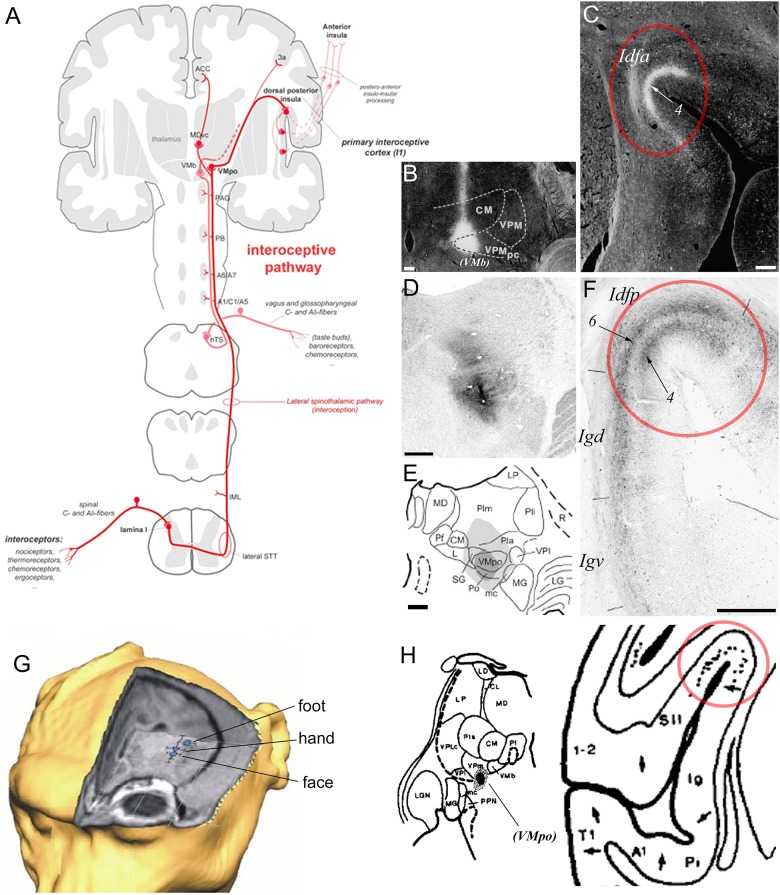
Primate ascending interoceptive pathway and primary interoceptive cortex. **(A)** Schematic representation of the ascending interoceptive pathway in primates. Inspired from Craig (2003b). **(B**,**C)** Darkfield photomicrographs of coronal sections in a rhesus macaque monkey brain, showing an autoradiographic injection site of tritiated amino acids in VMb (labeled here “VPMpc”), and its resulting anterograde labeling in Idfa (Pritchard et al., 1986). The labels “(VMb)”, “Idfa” and “4” were added by the present author. **(D**–**F)** Brightfield photomicrographs and chart of coronal sections in a cynomolgus macaque monkey brain showing an immunoreacted injection site of cholera toxin b centered in VMpo **(D**,**E)** and its resulting anterograde labeling in layer 4 in Idfp (Craig, 2014). Retrograde labeling also occurred in Idfp layer 6 and the adjacent area Igd. **(G)** 3D representation of the estimated localization of the insular thermal laser-evoked potential (LEP) sources for the stimulation of the head, hand, and foot in one cynomolgus macaque monkey (Baumgartner et al., 2006). **(H)** Plot of an injection site of tritiated amino acids in a small thalamic region corresponding to VMpo, in between VPI and VMb, in case RM17R (left), and distribution of the anterograde labeling produced by this injection in the dorsal fundus of the insular cortex, a region corresponding to Idfp (right) (Burton and Jones, 1976). The label “(VMpo)” was added by the present author. All illustrations in the panels of this figure were adapted with permission from the publishers of the cited references.

The region straddling the SPS or dorsal fundus of the insular cortex constitutes the primary cortical terminus of a primate specific interoceptive afferent pathway representing the ongoing physiological status of the organs and tissues of the body (Craig, 2002, 2003a; Figure 4A). This interoceptive pathway begins with the peripheral innervation of the organs and tissues of the body (e.g., skin, gut, heart, and lungs) by the bare axon terminals (or “interoceptors”) of unmyelinated C or thinly myelinated Aδ primary afferent fibers. Following their ascending course in the peripheral spinal or cranial nerves, these fibers terminate mainly in the lamina I of the spinal cord (SpL1) and in the bulbar solitary tract nucleus (nTS), respectively. Both regions project to various homeostatic and pre-autonomic centers in the spinal cord and brainstem, including the intermediolateral cell column, parabrachial nucleus, locus coeruleus, and periaqueductal gray (Beckstead et al., 1980; Craig, 1993, 1995a). In primates, the projections of SpL1 and nTS extend further anteriorly to the thalamic VMpo and VMb, respectively (Beckstead et al., 1980; Craig et al., 1994; Blomqvist et al., 2000; Craig, 2002, 2014). Rather than projecting to the classical primary somatosensory cortex, VMpo and VMb project primarily to the dorsal fundus of the insular cortex, with VMpo projecting to Idfp and VMb to Idfa (Benjamin and Burton, 1968; Pritchard et al., 1986; Craig, 1995b, 2014). As illustrated in Figure 4B–F, the mediolateral boundaries of the projections from VMb (Figure 4B,C) and VMpo (Figure 4D–F) to the fundus are abrupt; they match exactly the boundaries of the distinct granular architectonic areas that we recognize as Idfp and Idfa. Prior, but yet unexplored, tract-tracing evidence suggests that VMpo and VMb project to a secondary site within the insula, overlapping approximately with the mound dysgranular area and the lateral agranular area, respectively (not illustrated) (Pritchard et al., 1986; Craig, 2014). The functional attributes of this secondary projection zone within the insula remain to be examined. In addition to the insula, VMpo, and VMb also contribute small projections to the somatosensory area 3a in the fundus of the central sulcus, and, finally, SpL1 contributes inputs to a small ventral caudal portion of the mediodorsal nucleus of the thalamus (MDvc), which projects to the fundus of the cingulate cortex (Figure 4A; Craig, 2002). The fact that the interoceptive pathway terminates invariably in cortical fundi has led to the idea that the interoceptive thalamocortical tract serves as a developmental anchor for the operculation of the insula and of the cingulate and central sulci (Craig, 2010, 2014; Evrard et al., 2014). These cortical fundi are crucial in early development and their vital homeostatic function perhaps requires rather short-distance connections to compensate for the relatively slower conduction of the initial C and Aδ fibers, as compared to the Aα and β fibers associated with the somatosensory cortex.

Published and preliminary electrophysiological and imaging works in the macaque monkey showed that the posterior dorsal fundus of the insula, Idfp, responds to innocuous and noxious thermal stimuli as well as to noxious mechanic pinch stimuli, with a somatotopic representation of the foot, hand, and face from posterior to anterior (Figure 4G; Baumgartner et al., 2006; Craig, 2010, 2011; Hartig et al., 2017). Taste stimuli in awake monkeys have been shown to activate neurons in the anteroposterior half of the dorsal fundus, corresponding to Idfa (Yaxley et al., 1990) and recent imaging studies specified that this activation is predominantly in the caudal end of Idfa (Idfa-c in Figure 3; Hartig et al., 2017, Hartig et al., 2018). An earlier microstimulation study showed that stimulation of a restricted region in the vicinity of this orofacial region produced vocal cord movement (Sugar et al., 1948), suggesting the presence of a complex representation of the phonatory organs overlapping with or anterior to the orofacial (feeding?) region (Evrard, 2018). Our ongoing examination of the rostral end of Idfa (Idfa-r) suggests that it may contain a deeper visceral representation. However, we suspect that visceral representation occurs also beyond Idfa-r, in a complex organization throughout the fundus.

Studies in humans showed that areas likely homologous to Idfp in the dorsal posterior fundus of the insula respond to graded cooling (Craig et al., 2000; Maihofner et al., 2002) and to graded noxious heat stimulation (Iannetti et al., 2005; Keltner et al., 2006; Segerdahl et al., 2015). The anteroposterior topography identified in the macaque (Craig, 2004, 2011, 2014) has also been demonstrated in the human dorsal posterior insula using cooling-specific (Hua et al., 2005), nociceptive-specific (Brooks et al., 2005; Henderson et al., 2007; Baumgartner et al., 2010), and C-tactile (Bjornsdotter et al., 2009) stimulation. Similar evidence indicates that the primary cortical area for gustatory and vagal afferent activity is located immediately anterior to the region activated via VMpo (Wang et al., 2008; Small, 2010); consistent with the presence of a homologous, coherent anteroposterior representation of all interoceptive activity. A detailed mapping of the sensory afferent projections of the primary interoceptive cortex is still clearly needed; it will have major implications for our understanding of the neurobiological and physiological grounds of emotional embodiment (James, 1994; Nummenmaa et al., 2014).

Despite accumulating evidence, the anatomical and functional identification of the dorsal fundus of the primate insula as a primary sensory cortex for interoception remains a matter of debate. In many prior anatomical studies, an architectonic border was drawn medial to the center of the fundus of the lateral sulcus (Rose, 1928; Roberts and Akert, 1963; Jones and Burton, 1976; Mesulam and Mufson, 1982a), as we drew for Idfp/a (Evrard et al., 2014). However, in other studies, the fundus was lumped together with the secondary somatosensory representation in the parietal operculum and, based upon recordings and tracing studies (Burton and Jones, 1976; Friedman et al., 1980; Robinson and Burton, 1980), were interpreted to indicate the presence of an anteroposterior somatotopy (head-to-foot). These studies were, by necessity, re-interpreted (Whitsel et al., 1969; Burton et al., 1995; Krubitzer et al., 1995), and modern studies have confirmed the presence of two main mechanoreceptive representations in the operculum of monkeys (S2 and PV) and humans (OP1 and OP4), which are both somatotopically organized from lateral (face) to medial (foot) across the parietal operculum, lateral to (and distinct from) the fundus of the superior limiting sulcus (SLS) of the insula (Krubitzer et al., 1995; Eickhoff et al., 2007). Tracer injections in the primary somatosensory cortex that produced well-delimited labeling straddling the dorsal fundus of the insula (i.e., our Idfp) also produced labeling in the posterolateral thalamus, where we now recognize VMpo, which strongly suggests a spread of the injection to area 3a (Friedman et al., 1980). Similarly, the stereotaxically placed tracer injection in “posterior VPM” that produced well-delimited labeling in the dorsal fundus that was denoted as the face representation of S2 (Figure 4H; Burton and Jones, 1976) was located between VPI and VMb at the posterior end of VPM, where VMpo can easily be misidentified as VPM, as described earlier (Blomqvist et al., 2000; Craig and Blomqvist, 2002; Craig, 2004).

### Posterior Granular Areas (Igd and Igv)

**Figure 5:**
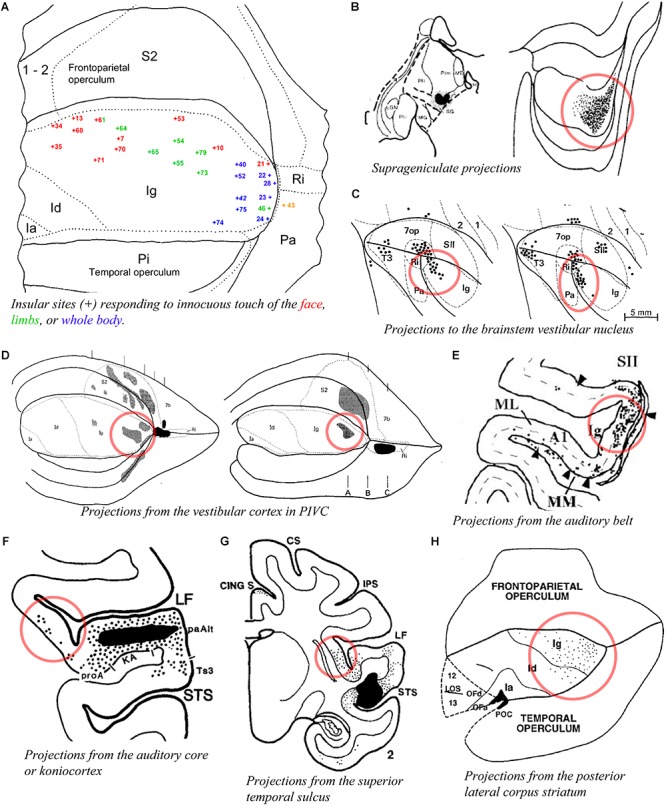
Activity and connectivity of the posterior granular areas of the macaque insula. **(A)** Flat map drawing of the insular cortex and adjacent opercula summarizing the localization (+ symbol) of microelectrode recording sites with single- or multi-unit responses to innocuous somatic stimuli on the face (red) or limbs (green), or of the whole body with, in some cases, a simultaneous response to auditory stimuli (blue) in the rhesus macaque monkey (*n* = 3). We consider that the dorsal region (red and green) corresponds very likely to our dorsal dysgranular area, the posterior region (blue) to our posterior granular areas (see text) (Schneider et al., 1993). **(B)** Plot of an injection site of tritiated amino acids (TAA) in a small thalamic region corresponding to the suprageniculate nucleus (SG) in case RM19L (left) and distribution of the anterograde labeling produced by this injection in the posterior end of the insula, likely corresponding to our posterior granular areas, Igd, and Igv (Burton and Jones, 1976). **(C)** Flat map plot of retrograde labeling in the posterior insula in cases M1 (left) and M2 (right) with an injection of horse-radish-peroxidase (HRP) in the brainstem vestibular nucleus (Akbarian et al., 1994). **(D)** Flat map plot of anterograde labeling in the posterior insula in cases RM4 (left) and RM3 (right) with an injection of TAA in the retroinsular (vestibular) cortex (Friedman et al., 1986). **(E)** Flat map plot of retrograde labeling in the posterior insula in cases MC with an injection of fast blue in area CM of the auditory belt (Smiley et al., 2007). **(F)** Coronal plot of retrograde labeling in the posterior insula with an injection of FB in the koniocortex-primary auditory area (KA) of the auditory cortex (Cipolloni and Pandya, 1989). **(G)** Coronal plot of anterograde labeling in the posterior insula with an injection of TAA in the superior temporal sulcus (STS). **(H)** Flat map plot of retrograde labeling in the posterior insula in case MS56 with an injection of WGA-HRP in the posterior lateral corpus striatum (Seltzer and Pandya, 1991). All illustrations in the panels of this figure were adapted with permission from the publishers of the cited references.

A prior physiological study obtained responses to whole body stimulation and to auditory stimuli in the posterior insula of the macaque monkey (Schneider et al., 1993; blue numbers in Figure 5A). Another study reported responses mostly to high-threshold or deep somatic stimuli in the dorsal posterior granular insula of the New World titi monkey, with auditory or mixed auditory and somatosensory responses located more ventrally (Coq et al., 2004). Tracing experiments showed that the posterior granular insula receives a dense input from the suprageniculate nucleus in the posterior thalamus (Burton and Jones, 1976; Figure 5B), which receives projections from the deep layers of the superior colliculus (Benevento and Fallon, 1975), superior temporal sulcus (Yeterian and Pandya, 1989), auditory belt and parabelt (Hackett et al., 1998), and frontal eye field (Huerta et al., 1986), as well as some projections from the spinal cord (Craig, 2004). The posterior granular insula projects to the brainstem vestibular nuclei (Figure 5C; Akbarian et al., 1994), and is strongly interconnected with the primary vestibular cortex (parieto-insular vestibular cortex, PIVC) which is located directly posterior to the insula in the retroinsular region (Figure 5D; Mesulam and Mufson, 1982b; Friedman et al., 1986; Guldin et al., 1992) and with other cortical vestibular processing sites (Mesulam and Mufson, 1982b; Akbarian et al., 1994). Similar to the suprageniculate nucleus, the posterior granular insula is connected with the auditory belt (Figure 5E; Smiley et al., 2007) and auditory core (Figure 5F; Cipolloni and Pandya, 1989), and with different portions of the poly-sensory upper bank of the superior temporal sulcus (Figure 5G; Seltzer and Pandya, 1991, 1994).

These findings are consistent with the original physiological evidence that the macaque’s PIVC occupies the retroinsular region of the LF and extends into the adjacent posterior granular insula (Grusser et al., 1990a,b). The posterior granular insula in monkeys integrates not only vestibular inputs but also proprioceptive, visual motion, and auditory activities (Chen et al., 2010; Shinder and Newlands, 2014), similar to the posterior end of the insula in humans (Baier et al., 2013; zu Eulenburg et al., 2013; Frank et al., 2014). This poly-modal integration likely contributes to “higher” vestibular functions (Oh et al., 2018), including self-motion perception in a body- (proprioceptive) and world- (audio-visual) centered referential system that could be organized into a dorsoventral gradient in Igd and Igv, respectively. Additional tracing evidence indicates connections with the dorsal portion of the dorsolateral striatum (Figure 5H; Chikama et al., 1997) and with the intermediate portion of the lateral dorsal nucleus of the amygdala (Ldi) (Stefanacci and Amaral, 2000), which could underlie the functional implication of vestibular activities in emotions (Lopez, 2016). In fact, in addition to the suggestion of a role in the monitoring of self-motion, the intimate role of vestibular activity in autonomic function provides a very reasonable hypothesis that deserves full consideration (Balaban and Porter, 1998), particularly in light of the tight structural relationship of PIVC with primary interoceptive cortex and the parallel interconnections they both share with the fundus of the central sulcus and the fundus of the cingulate sulcus.

## Dysgranular Insula

In our architectonic study, we divided the classical dysgranular sector into four areas and we subdivided three of these areas (Idd, Idm, and Idv) into thin horizontal stripes (Figure 2A; Evrard et al., 2014). Recent tract-tracing in our laboratory demonstrated an optimal overlap between the architectonic boundaries of the areas or modules and the hodological boundaries defined by the distinct patterns of anterograde or retrograde labeling produced in the insula with injections of tracers elsewhere in the brain, for example, in distinct areas of the prefrontal cortex (Krockenberger et al., unpublished). This overlap validates the novel architectonic parcellation (Evrard et al., 2014) and suggests that each stripe of the insula has specific neuronal connections and functions. Ongoing analyses aimed at determining the connectivity pattern of each stripe will help elucidate the functional integration occurring across the dysgranular insula and drive the actual functional model of the insula toward a refined version with new testable hypotheses. In the meantime, the paragraphs below review the evidence for the distinction between a dorsal “intrapersonal” field and a mound-ventral “interpersonal” field.

### Dorsal Dysgranular Areas (Idd)

**Figure 6:**
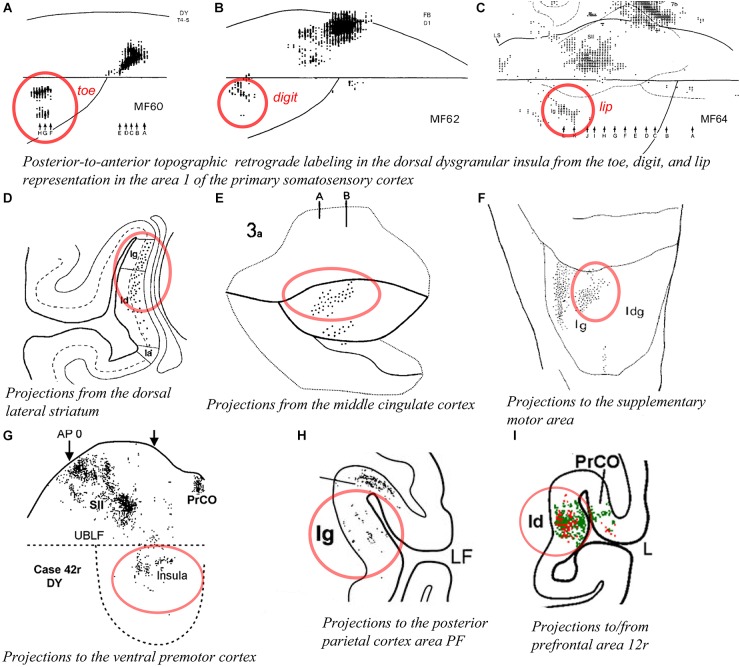
Connectivity of the dorsal dysgranular area of the macaque insula. In all panels, the regions of labeling in the dorsal dysgranular region of the insula are denoted with a red circle. **(A**–**C)** Retrograde labeling produced in the posterior region of the dorsal part of the dysgranular insula with an injection of **(A)** diamidino yellow in the “toes 4–5,” **(B)** fast blue in the “digit 1,” and **(C)** diamidino yellow in “lower lip” representations in area 1 of the primary somatosensory cortex in cases MF60, MF62, and MF64, respectively (Burton et al., 1995). **(D)** Plot of retrograde labeling in a coronal section of the insula produced in case MS51 with an injection of WGA-HRP in the dorsal lateral portion of the corpus striatum. The authors considered as “granular” (Ig), a small region of the insula that was likely part of what we consider the dorsal dysgranular area (Chikama et al., 1997). **(E)** Flat map plot of anterograde labeling in the insula in case 3a with an injection of tritiated amino acid in the middle cingulate sulcus (Mesulam and Mufson, 1982b). **(F)** Flat map plot of retrograde labeling in the insula in case 3 with an injection of fast blue in the supplementary motor area (SMA or F3). The authors considered as “granular” (Ig) the region of the insula that we consider as the dorsal dysgranular area (Luppino et al., 1993). **(G)** Flat map plot of retrograde labeling in the insula in case 42r with an injection of diamidino yellow in the ventral premotor cortex (PMv or F5) (Gerbella et al., 2011). **(H)** Coronal plot of retrograde labeling in the insula in case 29 with an injection of diamidino yellow in the area PF of the posterior parietal cortex. The authors considered as “granular” (Ig) the region of the insula that we consider as the dorsal dysgranular area (Rozzi et al., 2006). **(I)** Coronal plot of retrograde (red) and anterograde (green) labeling in the insula in case 44r with an injection of lucifer yellow in the area 12r of the prefrontal cortex (Borra et al., 2011). All illustrations in the panels of this figure were adapted with permission from the publishers of the cited references.

As mentioned earlier, Schneider et al. (1993) found units responsive to innocuous tactile stimulation of the contralateral head, trunk, hand, and foot organized across the posterior two thirds of the anteroposterior extent of the dorsal dysgranular areas (Figure 5A; Schneider et al., 1993). Notably, their report suggests that the basic anterior-to-posterior (head-to-foot) somatic topography of the granular cortex in the dorsal fundus is preserved in the dorsal dysgranular areas. They stated that anterior units responded predominantly to stimulation of the face and mouth, while posterior units responded predominantly to stimulation of the limbs and trunk. Microstimulation of the dorsal fundus and adjacent dorsal dysgranular areas produced movements of the foot, hand or face that were also roughly organized from posterior to anterior (Sugar et al., 1948). A similar anteroposterior somatotopography in the dorsal dysgranular areas is suggested by the pattern of labeling produced by injections of anterograde or retrograde tracers in the primary and secondary somatosensory cortical areas (Mesulam and Mufson, 1982b; Friedman et al., 1986; Burton et al., 1995; Cipolloni and Pandya, 1999). For example, although interpreted otherwise by the authors, a retrograde tracer injection in the hindlimb representation in the primary somatosensory area 1 (S1) produced labeling in the posterior portion of the dysgranular region, dorsal to the mound (Figure 6A; Burton et al., 1995), whereas a similar injection in the “lower lip” representation of S1 produced labeling in the middle portion of the dorsal dysgranular areas (Figure 6C); and an injection in the “hand digit 1” region produced intermediate labeling (Figure 6B). In all three cases, the insular cortical labeling was organized in “distinct patches,” or anteroposterior slabs of neurons, and the location of the labeling would lie within the dorsal dysgranular area denoted in our map (Figure 3), and not in the granular insula area as denoted by the authors. In a separate study, recording sites overlapping with or anterior to the face representation in the aforementioned studies were shown to respond to baroreceptive and mechanoreceptive inputs (Zhang et al., 1999), indicating the multi-modal nature of the integration occurring in the dorsal dysgranular insula.

Tracing studies with injections in other cortical and subcortical structures generally also support the view that the dorsal dysgranular areas and the mound dysgranular areas have distinct connections. For example, “patches” or “slabs” of anterograde or retrograde labeling were produced in the dorsal dysgranular areas but only sparsely (or not at all) in the mound areas with tracer injections in the dorsolateral “sensorimotor” region of the striatum (Figure 6D; Chikama et al., 1997; Fudge et al., 2005), the “motor” posterior cingulate cortex (Figure 6E; Mesulam and Mufson, 1982b; Mufson and Mesulam, 1982; Morecraft et al., 2004), the supplementary motor area (Figure 6F; Luppino et al., 1993), distinct divisions of the F5 area in ventral premotor cortex (PMv or F5) (Figure 6G; Gerbella et al., 2011), distinct sectors of area 7b in the posterior parietal cortex (Figure 6H; Rozzi et al., 2006), the intermediate part of area 12r (Figure 6I; Borra et al., 2011), and the convexity of the rostral sector of area 46vc (not shown) (Gerbella et al., 2012). All of these structures are associated with higher-order proprioceptive processing and complex, motivated motor and behavioral planning, involving particularly the hand and mouth (Rizzolatti and Luppino, 2001; Haber, 2003; Borra et al., 2011). Several of these structures have direct projections to the cervical spinal cord (F3, F5, and PFG). Thus, the dorsal dysgranular areas are interconnected with Idfp/a, with somatosensory cortex, and with higher-order areas involved in behavioral execution. Together, these connections suggest that the dorsal dysgranular areas may be involved in integrating interoceptive activity with proprioceptive and mechanoreceptive inputs, involved in object-directed, motivated behavior. Considering a potential insular representation for “self-related” processes, a body-centered integration could play a role in the representation of self-agency, rather independently of the environmental context. In humans, lesions involving the dorsal portion of the insula have been associated with an alteration of the sense of body ownership that is necessary for self-agency (Karnath and Baier, 2010).

### Mound and Ventral Dysgranular Areas (Idm and Idv)

**Figure 7:**
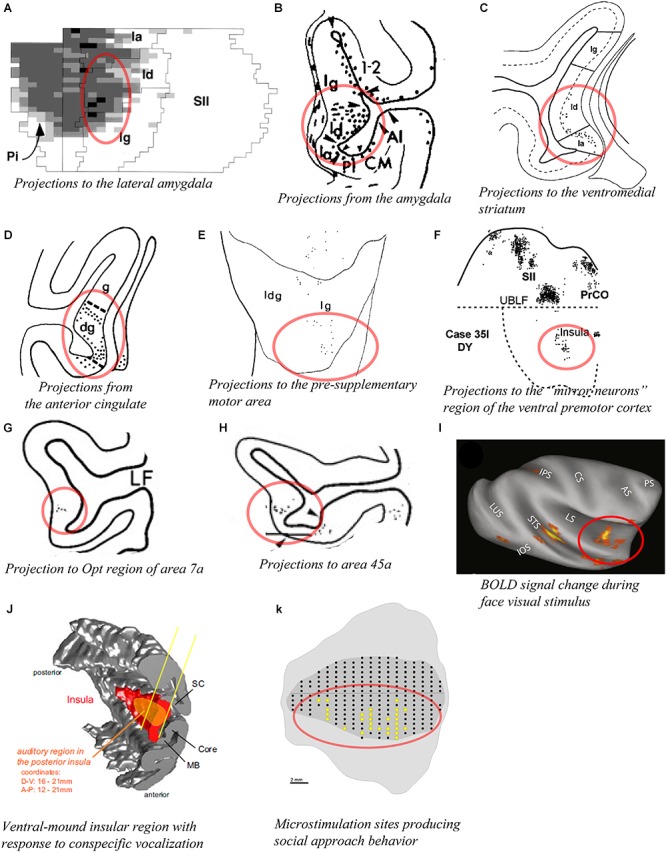
Connectivity of the mound and ventral dysgranular areas of the macaque insula. In all panels, the regions of labeling in the mound and ventral dysgranular regions of the insula are denoted with a red circle. **(A)** Flat map plot of fluorescent retrograde labeling in the insula in case M28-92R with an injection of fast blue in the intermediate division of the lateral nucleus. In the flat map, up is rostral and right is dorsal (Stefanacci and Amaral, 2000). **(B)** Coronal plot of anterograde labeling in the insula in case FCP-2 with an injection of tritiated amino acids in the amygdala (Amaral and Price, 1984). **(C)** Coronal plot of retrograde labeling in the insula in case MS14 with an injection of WGA-HRP in the ventromedial portion of the striatum (Chikama et al., 1997). **(D)** Coronal plot of anterograde labeling in the insula in case 2a with an injection of TAA in the anterior cingulate cortex (Mufson and Mesulam, 1982). **(E)** Flat map plot of fluorescent retrograde labeling in the insula in case 4l with an injection of fast blue in the pre-supplementary motor area. In the flat map, up is dorsal and right is posterior (Luppino et al., 1993). **(F)** Flat map plot of fluorescent retrograde labeling in the insula in case 35l with an injection of diamidino yellow in the “mirror neuron” sector of the ventral premotor cortex. In the flat map, up is dorsal and right is anterior (Gerbella et al., 2011). **(G)** Coronal plot of retrograde labeling in the insula in case 23 with an injection of WGA-HRP in the Opt region of area 7a (Rozzi et al., 2006). **(H)** Coronal plot of retrograde labeling in the insula in case 37l with an injection of fast blue in area 45a (Gerbella et al., 2010). **(I)** Lateral view of an inflated MRI brain volume showing areas with significant BOLD signal change response to face visual stimuli compared to other categories of non-face visual stimuli (fruit, house, and fractals) in one awake monkey. Similar results were obtained in a second awake monkey (Ku et al., 2011). **(J)** 3D reconstruction of the cortical mantel showing the localization of an insular region (orange) with a high density of neurons responding predominantly to vocalization stimuli among other non-vocalization auditory stimuli. This region spans from the posterior to mid insula and across the structural mound, visible here with the distinct convexity (mound) within the 3D rendering of the insula (Remedios et al., 2009). **(K)** Unfolded lateral view of the left insula in one macaque monkey (M1) showing the sites (yellow dots) from which electrical microstimulation evoked “affiliative” behavior (Caruana et al., 2011). In all panels, the structural region corresponding to the mound and ventral dysgranular areas is circled in red. All illustrations in the panels of this figure were adapted with permission from the publishers of the cited references.

The entire insula has widely distributed interconnections with several amygdalar nuclei, including the lateral nucleus, different divisions of the basal nucleus, and the central nucleus (Aggleton et al., 1980; Turner et al., 1980). However, the published evidence clearly suggests that the agranular and the dysgranular mound areas have considerably denser connections with the basal and lateral nuclei of the amygdala than do the dorsal dysgranular and granular areas. For example, retrograde labeling from the dorsal intermediate division of the lateral nucleus (Ldi) is predominantly concentrated in a ventral and anterior portion of the insula, corresponding to the agranular, and anterior mound areas (Figure 7A; Stefanacci and Amaral, 2000). Connections with the amygdala described in other studies are generally consistent with this interpretation (Figure 7B; Mufson et al., 1981; Amaral and Price, 1984). Recordings in the relevant portions of the amygdala revealed strong responses to images of emotional monkey faces, including some more responsive to identity, and some to emotion (Gothard et al., 2007; Mosher et al., 2010). Consonant with the pattern of connections with the amygdala, tracing studies also showed strong labeling in the structural region of the mound following retrograde tracer injections in the “limbic” ventromedial portion of the striatum (Figure 7C; Chikama et al., 1997; Fudge et al., 2005). In addition, patches or slabs of anterograde or retrograde labeling were produced in the mound with little or no labeling in the dorsal dysgranular areas following injections in the “limbic” anterior cingulate cortex (Figure 7D; Mesulam and Mufson, 1982b; Mufson and Mesulam, 1982), the pre-supplementary motor area (Figure 7E; Luppino et al., 1993), a subdivision of F5 (PMv) different from the ones that are interconnected with dorsal dysgranular areas (Figure 7F; Matelli et al., 1986; Gerbella et al., 2011), distinct subregions of area 7a other than those connected with the dorsal dysgranular area (Figure 7G; Rozzi et al., 2006), the caudal sector of area 46vc (not shown) (Gerbella et al., 2012), and area 45a (Figure 7H; Gerbella et al., 2010). Preliminary retrograde tracing in our laboratory showed that distinct subareas of the ventral and mound dysgranular areas also project to the lateral hypothalamus and the periaqueductal gray, indicating that this integrative region has efferent projections able to induce or regulate emotional behaviors (Saleh et al., 2017), as shown in microstimulation in the awake monkey (Caruana et al., 2011).

The functional associations of these particular higher-order regions suggest that the mound and ventral dysgranular areas might have a crucial role in behaviors involving the face and hands in relation to social communication. For instance, the pre-SMA area is associated with “signaling when a movement can be executed, according to the environmental context” (Luppino et al., 1993; Rizzolatti and Luppino, 2001). The portion of PMv (F5) connected with the mound areas contains the so-called “mirror” neurons that are active during the execution or observation of hand- and face/mouth-related actions (Gallese et al., 2004; Gerbella et al., 2011). Area 7a integrates sensorimotor, visual and auditory activities related to extra-personal space (Rozzi et al., 2006). The portion of area 46vc that is interconnected with the mound areas is also interconnected with the anterior cingulate and orbitofrontal cortices, and may be involved in organizing higher-order motor control in relation to internal states, motivation, and reward (Gerbella et al., 2012). Finally, area 45a has been associated with communication (Gerbella et al., 2010). Thus, the mound region of the insula could be involved in high-order integration of interoceptive, extra-personal (social and communicative), and limbic (amygdala and ventral striatum) activities in the context of socially relevant emotional behavior. Notably, activation in the monkey’s ventral insula has been associated with several different social behaviors, including “social challenge” and aggressive behavior (Rilling et al., 2004), exposure to conspecific and familiar faces (Figure 7I; Ku et al., 2011), and exposure to conspecific vocalizations (Figure 7J; Remedios et al., 2009). In addition, Jezzini et al. (2012) showed that electrical microstimulation of the ventral region of the insula corresponding to the mound and ventral dysgranular areas reduced the heart beat frequency and produced social approach behavior (Figure 7K; Caruana et al., 2011). Tracer injections in the microstimulated sites confirmed the aforementioned connections with the cingulate cortex, basal and lateral amygdala, ventral striatum, and lateral hypothalamus (Jezzini et al., 2015). The authors emphasized the “motor” role of the ventral insula and associated this region with social behavior, naming it an “affiliative field” (Jezzini et al., 2015), which is consistent with our “interpersonal/social” region (Evrard et al., 2014; Evrard and Craig, 2015).

## Agranular Areas

An extensive series of tracing studies of the orbital and medial prefrontal cortex (OMPFC) indicates that each of its 22 areas has distinct connections, grouping the areas into 2 networks, an orbital “viscerosensory” network that may be involved in the assessment of food stimuli (Price, 2007) and has been suggested to serve as a “primary sensory area for emotions” (Barbas et al., 2011); and a medial “visceromotor” or “emotional motor” network that has descending connections with visceromotor centers, such as, the anterior part of the lateral hypothalamus (LHa) and the midbrain periaqueductal gray (PAG) (Price et al., 1996; Price, 2007). Among the agranular insular areas identified anterior to the limen insula (L) (Carmichael and Price, 1994; Evrard et al., 2014), areas Iam, Iapm, and Ial are part of the orbital network. Iam receives olfactory input directly from the primary olfactory cortex (POC) and produces short-latency responses to stimulation of the olfactory bulb (Ongur et al., 1998). Area Iapm receives input from different parts of the olfactory complex and produces long-latency responses to stimulation of the olfactory bulb (Ongur et al., 1998); it seems to also receive thalamic input from the ventral (presumably visceral) part of VMb (Carmichael and Price, 1995b). Although this remains to be clarified, area Ial seems to receive similar projections from the ventral part of VMb, but no input from POC (Carmichael and Price, 1995b). All three agranular areas are also interconnected with different parts of the amygdala, particularly the basal nucleus (Carmichael and Price, 1995a). The “visceral/olfactory” area Iapm and “visceral” Ial are interconnected with the rostral ventromedial part of the basal nucleus, whereas the “olfactory” Iam is interconnected with a spatially separate cluster located more dorsally within the basal nucleus (Carmichael and Price, 1995a). All three areas are interconnected with the anterior cortical nucleus of the amygdala but, in an association that is reminiscent of the connections with the basal nucleus, only areas Iapm and Ial are interconnected with the medial nucleus (Carmichael and Price, 1995a).

In the model of Price et al. (1996), area Iai stands out as an “orbital component of the medial network” (Price, 2007). It projects strongly to the anterior part of the lateral hypothalamus (LHa) (Ongur et al., 1998), the ventrolateral part of the basal nucleus of the amygdala (Bvl) (Carmichael and Price, 1995a), and the PAG (An et al., 1998). Notably, the regions of LHa and Bvl that Iai projects to, in turn, project to the same region of PAG as Iai; and all three regions (LHa, Bvl, and PAG) have been shown to have a crucial role in autonomic and emotional behavioral responses. Area Iai also projects to the substantia nigra, ventral tegmental area, median raphe, and peripeduncular tegmental nucleus (An et al., 1998), which further supports the role of Iai in the “visceromotor” network. Microstimulation of the anterior agranular insula in the macaque monkey has produced both physiological changes with effects on cardiovascular and respiratory functions (Kaada et al., 1949; Kaada, 1951; Caruana et al., 2011; Jezzini et al., 2012) and behavioral effects reminiscent of disgust facial expression (Caruana et al., 2011; Jezzini et al., 2012).

Although the anatomical isolation of Iai from the other anterior insular areas appeared obvious in the prior contributions by Price and colleagues, a recent re-examination of the relevant tracing cases indicated some of the anterograde and, more particularly, retrograde labeling originally attributed to Iai may also belong to the adjacent area Ial. This redefinition of the areal localization of labeling became evident after the discovery of the VEN and FN in the anterior insula in the macaque monkey (Evrard et al., 2012). Indeed, as summarized in the next paragraph, the VEN and FN seem to be predominantly located in one architectonic area, Ial (Horn F.M. et al., 2017), and a re-examination of the Price cases using the VEN/FN localization as a landmark disclosed crucial projections of Iai to LHa, Bvl, and PAG that are, in fact, shared with Ial. Thus, in a slight revision of the model of Price and colleagues, both Iai and Ial could serve as an output stage with efferent control of autonomic functions (Figure 3).

## Von Economo and Fork Neurons

**Figure 8:**
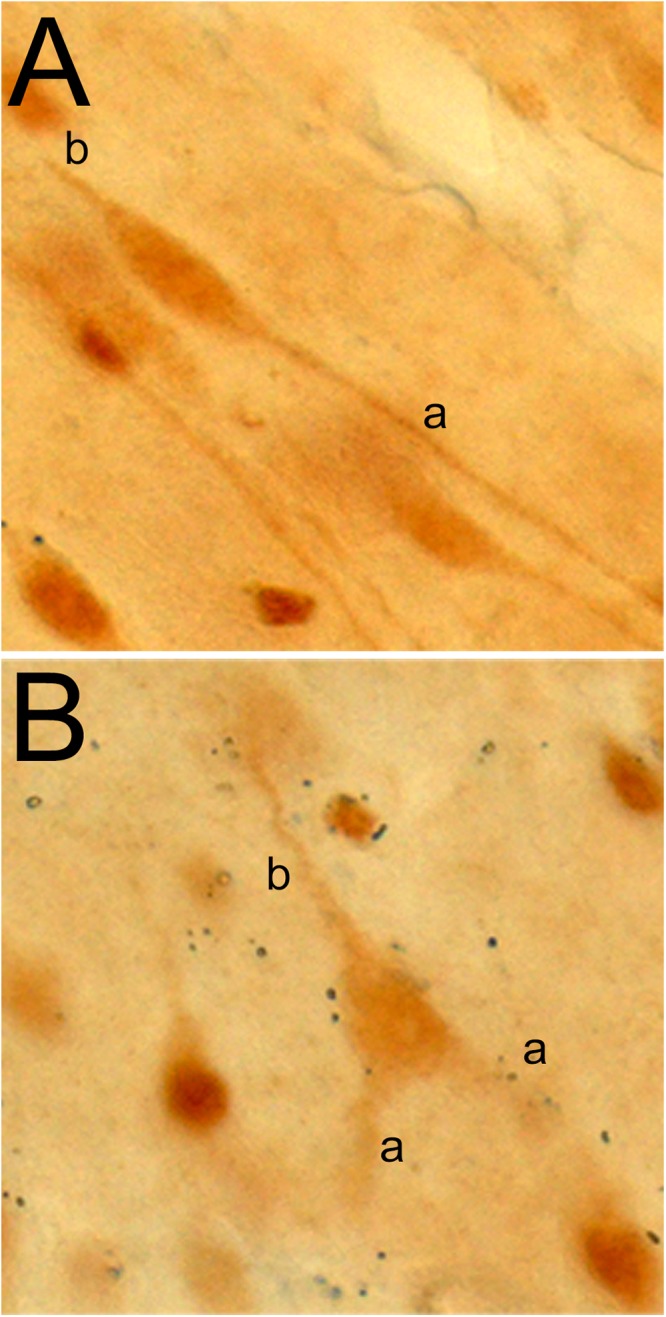
Photomicrographs of a VEN **(A)** and fork neuron **(B)** in the macaque monkey insula, immunostained with an antibody raised against the vesicular monoaminergic transporter 2 (VMAT-2; 1:1000, AB1598P, Millipore). The letters “a” and “b” in each panel refer to the apical and basal dendrites, respectively. Adapted with permission from Evrard (2018).

The von Economo neuron and fork neuron are morphologically specialized, or “atypical,” cortical projection neurons [as opposed to the “typical” pyramidal neuron (PN) (Nieuwenhuys et al., 2009)]. In primates and other mammalians, they are localized predominantly in the AIC and ACC (von Economo, 1926; Ngowyang, 1932). The VEN has a large spindle-shaped perikaryon with a single basal dendrite (instead of the classical basal tuft of the PN) that is as thick as the apical dendrite (Figure 8A). The FN, that accompanies the VEN, has a triangular perikaryon prolonged by bifid apical dendrites and a single basal dendrite (Figure 8B). Their exact respective functions remain to be elucidated, but they originally drew attention as a potential cellular contributor to the evolutionary emergence of human awareness (Allman et al., 2005; Craig, 2009). The VEN and FN are selectively degenerated in the early stage of neuropsychiatric disorders of selfhood, particularly including the behavioral variant of the frontal temporal lobe dementia (bvFTD) (Kim et al., 2011). Conversely, their presence is preserved in super-agers who maintain highly efficient cognitive, emotional, and social skills throughout aging (Gefen et al., 2015).

### Presence of the VEN and FN in Primate and Non-primate Species

While the VEN and FN were assumed to occur only in humans and great apes, among primates (Nimchinsky et al., 1999), both cells were identified in AIC and ACC in lesser apes (gibbon and siamang), and Old World monkeys including the rhesus and cynomolgus macaque monkeys (Stimpson et al., 2011; Evrard et al., 2012; Evrard et al., unpublished). In addition to primates, the VEN and FN occur in elephants, dolphins and whales, which are all highly encephalized and/or display complex social behaviors, suggesting a role in interoceptive processes supporting social behavior (Hof and Van der Gucht, 2007; Butti et al., 2009, 2011; Hakeem et al., 2009). More recently, the VEN and FN were observed in various cortical regions in pygmy hippopotamus, cow, sheep, deer, horse, pig, and rock hyrax- species closely related to cetaceans, or elephants (Raghanti et al., 2015). While some species, such as, the hippopotamus have a ubiquitous distribution of cells, other species, like the cow, have a relatively high concentration of VENs in a region analogous to AIC. The occurrence of the VEN and FN in so many species indicates that the VEN and FN are not restricted to large-brained or socially complex species. They may have repeatedly emerged throughout evolution in response to selective mechanisms that remain to be elucidated (Raghanti et al., 2015). The specific relation of the human VEN and FN to neuropsychiatric disorders, including bvFTD, and the neuronal projections of these cells in the macaque monkey (see below) emphasize their role in the autonomic regulation of cognitive processes in primates.

### Areal Localization of the VEN and FN

As in humans, the VEN and FN in macaques are present in layer 5b of the anterior agranular insula. They account for about 3 and 1% of the total number of local layer 5 neurons, respectively (Evrard et al., 2012). They are also observed in the ACC, however sparsely, as compared to the macaque AIC or human ACC. Rather than being dispersed throughout the anterior insula, the VEN and FN are consistently clustered together within a small region (Evrard et al., 2012). A close comparison of the localization of this cluster with the localization of the architectonic boundaries of the insula (Evrard et al., 2014) revealed that the VEN and FN occur exclusively in one architectonic area, the “Ial” or “VEN area” (Horn F.M. et al., 2017; Horn and Evrard, unpublished). This specific distribution suggests a common developmental, hodological and functional fate for the VEN and Ial. Thus, examining the area as a whole could provide major clues about the VEN and FN (e.g., their connections) that are otherwise difficult to obtain since they themselves represents only about 3 and 1% of the local neuronal population, respectively.

### VEN and FN Gene Expression and Projections

Just like in humans, the macaque VEN (and FN) expresses various genes that occur only in a small fraction of local PN. These genes include those coding for the neurofilament protein SMI-32, disrupted-in-schizophrenia-1 protein (DISC-1), dopamine D3 receptor, serotonin 2b receptor, vesicular monoamine transporter 2 (VMAT-2; Figure 8A,B), and gamma-aminobutyric acid (GABA) receptor subunit θ (GABRQ) (Allman et al., 2005; Evrard et al., 2012; Dijkstra et al., 2016). The macaque VEN and FN also express an isoform of the phosphate-activated glutaminase, confirming that they are glutamatergic excitatory neurons (Evrard et al., 2012).

In addition to the aforementioned genes, the VEN and FN express *FEZF2* and *CTIP2*, two transcription factors essential for the developmental specification of subcerebral (i.e., toward midbrain and brainstem) cortical projection neurons (Cobos and Seeley, 2015). As in humans, the VEN in macaques is volumetrically larger than the local PN, suggesting also long-distance projections (Evrard et al., 2012). Using tracer injections in the macaque AIC and ACC (Evrard et al., 2012), we recently confirmed an earlier hypothesis that the insular VEN projects to contralateral AIC and ipsilateral ACC (Allman et al., 2005; Craig, 2009). With injections in various subcerebral nuclei, we showed, however, that the bulk of the VEN projections target brainstem pre-autonomic nuclei, including the PAG and parabrachial nucleus (PBN), rather than cerebral cortex areas (Horn F. et al., 2017; Saleh et al., 2017; Chavez et al., unpublished).

These projections fit nicely with recent interoceptive predictive coding models proposing that the AIC infers, compares and updates interoceptive predictions, and that the descending projections of the VEN convey “interoceptive error attenuation” commands either via top-down sensory gating (PBN) or regulation of autonomic control (PAG), hence reducing interoceptive prediction error or “free-energy” (Critchley and Seth, 2012; Seth and Friston, 2016). The large volume and long-distance projections of the VEN and FN is also consistent with the role of AIC in “global workspace consciousness” (Dehaene et al., 2017). Notably, a recent examination of the connectivity of the VEN-containing region of the AIC in humans confirmed the existence of a robust functional relation to PBN; this functional connectivity was almost completely disrupted in coma patients (Fischer et al., 2016), suggesting a functional link between awareness and arousal, which have been proposed as being fundamental components of human consciousness (Laureys et al., 2009). In this context, the VEN and FN most likely support the function of local PNs, with their hypothetically faster columnar processing and projection, their impulse could precede the pyramidal firing and give an “edge” to the overall AIC function by “preparing” the target nucleus to imminent pyramidal inputs (Evrard, 2018).

## Possible Organizational Axes

### “Sensory-Motor Axis”

The present model and the overall architectonic sectorization of the insula from granular to agranular share several characteristics with the classical somatic sensory-motor cortices. The primary interoceptive cortex in the dorsal fundus of the insula is granular and receives high-resolution sensory inputs, like the primary somatosensory cortex in the post-central gyrus of the parietal lobe (Qi et al., 2008). The agranular AIC resembles the agranular primary motor cortex in the pre-central gyrus of the frontal lobe (M1). AIC receives distinct sensory thalamic inputs from the ventral part of VMb (Carmichael and Price, 1995b), just like M1 receives distinct spinal (laminae V-VII), and cerebellar inputs via the separate posteroventral part of the ventral lateral nucleus of the thalamus (VLpv) (Craig, 2006; Evrard and Craig, 2008). The denomination “motor” is partly misleading because both S1 and M1 receive sensory afferents, and may have been “superimposed” together early in evolution (Lende, 1963; Beck et al., 1996; Striedter, 2005). Both M1 and AIC also contain specialized neurons, the giant Betz cell in M1 and the large VEN and FN in AIC, respectively, located in layer 5, which project to distant subcerebral targets proximal to the effector organs. The giant Betz cells project to the motoneuron lamina of the ventral horn of the spinal cord and directly affect the control of striate muscle contraction. Likewise, the VEN and FN could project to pre-autonomic and perhaps pre-ganglionic nuclei that affect peripheral autonomic functions. The anterior agranular insula does not seem to have as fine-grained a topographic organization as the representation of ethological muscle synergies in M1 (Aflalo and Graziano, 2006); if any, its topographic organization remains to be elucidated. It likely mirrors the intricate organization of the solitary tract nucleus and motor nucleus of the vagus nerve (Beckstead et al., 1980). Finally, although somatosensory area 3b, which sits between granular S1’s areas 1-2 and agranular M1’s area 4, has been presented as dysgranular (Qi et al., 2008), the similarity between the single dysgranular area 3b and the numerous dysgranular stripes of the insula is not so obvious and limits the overall comparability. Nevertheless, the idea of a sensory-to-motor gradient in the insula (and possibly cingulate cortex) remains appealing and could represent an interocepto-autonomic counterpart of the classical haptic-locomotive S1-M1 system.

### “Spino-Cranial Axis”

One dimension that needs to be added to the sensory-motor axis relates to the anatomical sympathetic and parasympathetic autonomic branches of the autonomic nervous system. The dorsal fundus of the insula is topographically organized with a posterior-to-anterior representation of spinal (foot-to-hand), trigeminal (head/face), and vagal (taste/viscera) inputs. The available tract-tracing and functional evidence indicates that this spino-cranial plan is reproduced across the different dysgranular stripes. A recent model suggested that the spinal and cranial afferents to Idfp and Idfa represent the ascending counterpart of the anatomically defined sympathetic (essentially thoracolumbar) and parasympathetic (essentially cranial with additional sacral components) branches of the autonomic nervous system, respectively (Craig, 2015). An earlier tracing study showed that corticospinal and corticobulbar projection neurons are distributed preferentially in the posterior and anterior halves of the insula, respectively (Keizer and Kuypers, 1984). Our current model proposes that the entire dysgranular activity is “funneled” anteriorly in the agranular insula. In such case, tracing and functional experiments are still needed to identify whether the respective efferent counterparts of the posterior “spinal” and anterior “cranial” dysgranular representations are processed in distinct or overlapping parts of the agranular insula. In short, are the insular neurons projecting to sympathetic and parasympathetic pre-autonomic and pre-ganglionic nuclei grouped together or are they spatially separated in distinct (anterior) insular areas and subareas? One additional level of complexity relates to the possible left-right asymmetric organization of autonomic efferent neurons in the anterior insula (Craig, 2005). Prolonged electrical microstimulations of the left and right anterior insula in humans, respectively decreased and increased heart rate frequency and blood pressure (Oppenheimer et al., 1992; Oppenheimer, 2006, 2007), suggesting a lateralized representation of parasympathetic (left) and sympathetic (right) functions. We know so far that the VEN and FN are more numerous in the right than in the left anterior insula (Allman et al., 2010; Evrard et al., 2012). Whether these and other neurons projecting to parasympathetic vs. sympathetic pre-autonomic and pre-ganglionic brainstem nuclei are distributed with an asymmetric preference needs to be examined as well.

### “Cognition-Emotion Axis”

Cognition and emotion have been traditionally separated, although such conceptual separation likely misses the evolutionary, developmental and neuroanatomical intimacy of the neurobiological mechanisms underlying “cognition,” and “emotion” (Pessoa, 2008). Functional imaging studies and meta-analyses have proposed the existence of distinct cognitive and emotional fields in the limbic cortex, with a dorsal cognitive and a ventral emotional field in both ACC (Medford and Critchley, 2010) and AIC (Kurth et al., 2010b). Imaging and microstimulation studies in the macaque monkey suggest the existence of a similar dorsoventral division in the AIC, with the dorsal AIC being associated with cognitive tasks (Wang et al., 2015) and the ventral AIC with emotional responses (Caruana et al., 2011). Notably, both the posterior granular areas and the dysgranular areas contain a dorsal proprioceptive/body-centered/egocentric region (Igd and Idd) and a ventral proprio-audio-visual/world-centered/allocentric region (Igv and Idm-Idv) (Figure 3). These dorsal and ventral regions are in direct anatomical continuity with the dorsal agranular areas (Iapl) and ventral agranular areas (Ial-Iai-Iam) (Figure 3), which overlap with the dorsal “cognitive” and ventral “emotional” regions defined in the prior functional studies (Caruana et al., 2011; Wang et al., 2015), respectively. This continuous dorsoventral “division,” which shifts from ego-/allo-centric, posteriorly, to cognition/emotion, anteriorly, suggests a mechanistic relationship that, if true, could prove insightful for our understanding of the neurobiological correlate of cognition and emotion. Compared to humans, the evidence for emotional and complex cognitive activities in the AIC in the macaque monkeys is rather scarce (Caruana et al., 2011; Wang et al., 2015). The emotional and cognitive functions of the macaque AIC could be related, in fact, to relatively basic feeding behaviors (Price, 2007). Although both human and non-human primates have an anterior agranular sector, the disproportionate expansion of AIC in humans (Bauernfeind et al., 2013) suggests that it might host more refined and diversified representations of complex and abstract embodied functions, reflected in the vast diversity of tasks activating the AIC in fMRI studies (Craig, 2009), including the recognition of oneself in the mirror (Devue and Bredart, 2011), a test that macaque monkeys fail to pass in the original form of the mirror experiment (Anderson and Gallup, 2011).

## Conclusion

Much remains left to do to understand how the insula is anatomically organized, processes interoceptive, metabolic and poly-modal activities, and possibly contributes to the emergence of subjective feelings in humans. The present working model of the insula in the macaque monkey resembles prior meta-analytic models of the human insula (Kurth et al., 2010b) and offers a framework for the development of testable functional hypotheses, with clear anatomical landmarks. The systematic exploration of these hypotheses will help refine the current model and shed new light on the neurobiological and evolutionary logic underlying the multiple functions attributed to the insular cortex in both non-human and human studies. One important question will be whether the distinct dysgranular subareas and agranular areas contain approximate repetitions of the same maps, in which multiple “functions” overlap each other, or whether they each represent predominantly separate insular functions. Addressing this question may help understand, for example, whether distinct emotional feelings are encoded together in the same areas by distinct groups of intermingled neurons, or whether they are encoded in spatially separate areas and subareas.

## Author Contributions

The author confirms being the sole contributor of this work and has approved it for publication.

## Conflict of Interest Statement

The author declares that the research was conducted in the absence of any commercial or financial relationships that could be construed as a potential conflict of interest.

## Footnotes

1 The absence of macroscopic separation and the intimate connectivity between the insula and the orbital prefrontal cortex in the macaque monkey blurs the exact delimitation between these two regions. In our work, we consider the agranular areas anterior to the limen, posterior to area 13 and adjacent to the anterior end of the claustrum, as being part of the insula, both in humans and monkeys. In humans, this region, which contains von Economo and Fork neurons, is clearly encased by the folding surrounding the insula, including the anterior peri-insular sulcus. In the macaque monkey, there is no clear macroscopic delineation, but the presence of von Economo and Fork neurons in a recognizable macroscopic structure anterior to the limen, posterior to area 13, and adjacent to the anterior end of the claustrum leads us to also consider this region as being part of the insula. Future developmental works, such as, those recently published by Gonzalez-Arnay et al. (2017) may help elucidate the regional affiliation of the frontoinsula.

## Abbreviations

AC: accessory gyrus of the insular
APS: anterior peri-insular sulcus
AR: anterior insular ridge
CS: central insular sulcus
FI: frontoinsula
FN: fork neuron
H: temporal gyri of Heschl
Iai: intermediate agranular area of the insula
Ial: lateral agranular area of the insula
Iam: medial agranular area of the insula
Iapl: posterior lateral agranular area of the insula
Iap1-2: posterior agranular area of the insula, subareas 1 and 2
Iapm: posterior medial agranular area of the insula
Idd1-5: dorsal dysgranular area of the insula, subareas 1 to 5
Idfa: anterior (granular) area of the dorsal fundus of the insula
Idfp: posterior (granular) area of the dorsal fundus of the insula
Idm1-3: mound dysgranular area of the insula, subareas 1 to 3
Idv1-2: ventral dysgranular area of the insula, subareas 1 and 2
IPS: inferior peri-insular sulcus
Ivfa: anterior (agranular) area of the ventral fundus of the insula
Ivfp: posterior (dysgranular) area of the ventral fundus of the insula
L: limen insulae
l1-2: long gyrus 1 and 2 of the posterior lobule of the insula
LF: lateral fissure
PN: pyramidal neuron
s1-3: short gyrus 1 to 3 of the anterior lobule of the insula
SG: suprageniculate nucleus of the thalamus
SPS: superior peri-insular sulcus
VEN: von Economo neuron
VMb: basal part of the ventromedial nucleus of the thalamus
VMpo: posterior part of the ventromedial nucleus of the thalamus.
